# Investigating the causal relationship between human blood metabolites and pulmonary hypertension: a two-sample Mendelian randomization study

**DOI:** 10.3389/fcvm.2024.1304986

**Published:** 2024-10-15

**Authors:** Xingdu Zeng, Bin Zhong

**Affiliations:** Department of Respiratory Medicine, The First Affiliated Hospital of Gannan Medical University, Ganzhou, Jiangxi, China

**Keywords:** serological metabolites, Mendelian randomization, pulmonary hypertension, instrumental variables, causal inference

## Abstract

**Background:**

Several recent investigations have posited that distinct metabolites in the bloodstream may be correlated with the pathogenesis of Pulmonary Hypertension (PH). Nonetheless, the interrelationship between the pathogenesis of PH and metabolite fluctuations remains incompletely elucidated, and findings may differ across studies.

**Methods:**

In the extant research, data from 486 metabolite-and PH-related genetic variants in human subjects were procured based on Genome-Wide Association Studies (GWAS) and Finnish databases. Univariate Mendelian Randomization analyses were deployed to evaluate the causal relationships between them. The utilization of the randomized Inverse Variance weighted(IVW) method served as the primary analytic framework in this Mendelian Randomization (MR) study. Additionally, four alternative computational strategies, encompassing MR-Egger, were employed as auxiliary methods. A myriad of tests, including Cochran's Q Test, MR-Egger intercept test, MR-PRESSO, leave-one-out analysis, and linkage disequilibrium score were incorporated to assess the robustness of the study outcomes. Metabolite pathway analysis was also executed to identify potential metabolic pathways.

**Results:**

After a series of validations and corrected for False discovery rate (FDR), we found a significant association between 1,5-anhydroglucitol (OR = 2.00, 95% CI: 1.39–2.89, *P* = 0.0002) and PH, and a significant association between pyridoxalate (OR = 0.59, 95% CI: 0.43–0.81, *P* = 0.0009) and 1-a achidonoylglycerophosphocholine (OR = 1.78, 95% CI: 1.22–2.58, *P* = 0.0026) had a suggested association with PH. In addition, the vitamin B6 metabolic pathway was also determined to be associated with PH.

**Conclusion:**

Conclusively, we isolated 1,5-anhydroglucitol, 1-arachidonoylglycerophosphocholine, and pyridoxate as causally implicated in PH, thereby proffering substantial theoretical substantiation for the formulation of future PH prevention and screening paradigms.

## 1 Introduction

Pulmonary Hypertension (PH) is a scarce yet grave cardiopulmonary ailment manifested by pathological alterations in pulmonary artery wall thickening, thrombosis, vascular remodeling, and tissue hypoxia. These culminate in increased pulmonary arterial resistance, inhibited blood flow, and ultimately augmented cardiac strain and cardiac failure (1, 2). Further progression of pulmonary heart disease can ensue. The mortality rate among patients afflicted with PH remains elevated despite extant advancements in medical technology. The 5-year survival rate for patients with PH has been documented to be approximately 57.3%, and the 10-year survival rate at approximately 37.3% (3), which signifies that early diagnosis and prevention for patients with PH are imperative. Currently, the etiology of PH remains inadequately elucidated, yet it may be correlated with genetics, environmental variables, pharmacological exposure, and viral infections (4–6).In the absence of straightforward and dependable diagnostic modalities for the timely identification of early-stage PH, approximately 21% of patients experience a protracted delay, exceeding two years, from symptom onset to accurate diagnosis (7). Despite the availability of multiple diagnostic tools for PH evaluation, such as right heart catheterization (RHC), often touted as the gold standard, and echocardiography, a ubiquitous clinical method (8), RHC is disproportionately costly and intrusive, leading to adverse physical and socioeconomic outcomes for the patient. Moreover, echocardiographic assessments of pulmonary artery systolic pressure are intrinsically subject to operator bias. Consequently, notwithstanding these established diagnostic paradigms, there remains a need to ascertain novel indicators for early prediction and potentially definitive diagnosis.

In subsequent years, a rising number of studies have been initiated to diagnose and monitor diseases by analyzing variations in organismal metabolites via metabolomics (9). PH frequently coincides with perturbations in serum metabolites; notably, polyamine, histidine, and sphingomyelin metabolic pathways have been associated with the severity of the condition in patients with PH (10).Additionally, eight metabolites implicated in lipid metabolism and fatty acid oxidation—such as lysophosphatidylcholine, phosphatidylcholine, perillic acid, and eight other metabolites—have been robustly linked to PAH progression (11). Furthermore, trimethylamine N-oxide (TMAO) and oxide metabolites exert a significant influence on the pathological trajectory of PH (12, 13). While these population-based, observational studies have identified a plethora of metabolites correlated with PH, it remains challenging to eliminate potential confounding variables or to circumvent limitations such as specimen volume. Therefore, additional exhaustive and pertinent research endeavors are warranted to discern biomarkers correlating with PH, which are clinically consequential for its early diagnosis.

Mendelian randomization (MR) constitutes a nascent methodology in genetic epidemiology. By nullifying potential confounders and reverse causality, it offers unique advantages for assessing complex diseases. Explicitly, MR investigations rely on GWAS datasets to procure genetic variant data as instrumental variables(IVs), facilitating effective evaluations of causal relationships between exposure and outcome. The current MR study employed 486 serum metabolites as exposure variables and PH as the outcome, thereby formulating hypotheses regarding their causal interrelationships and inaugurating new pathways for the exploration of PH biomarkers and potential metabolic pathways.

## 2 Methods

### 2.1 Experimental design program

**Figure 1:**
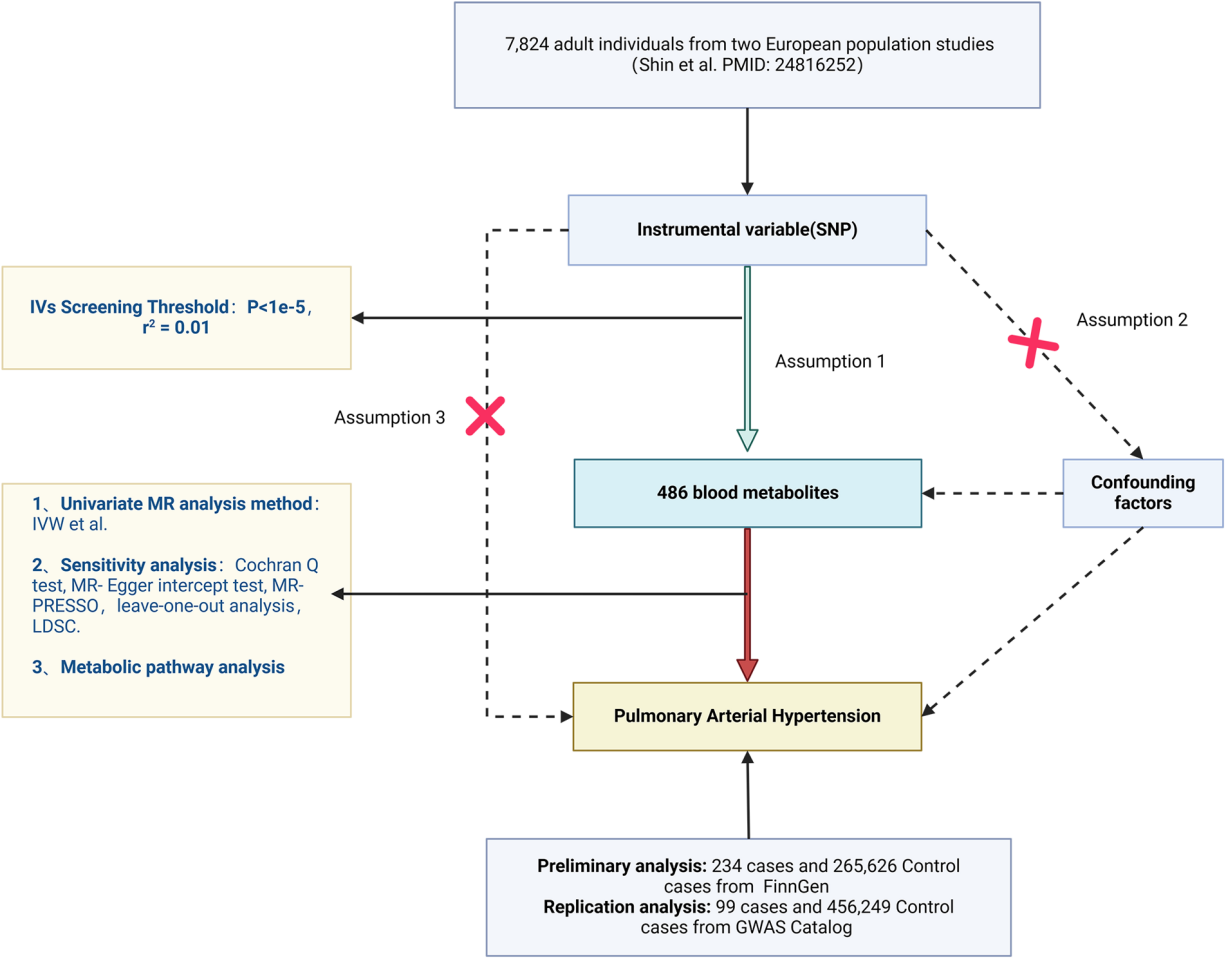
Flowchart of the design of this study.

In the extant study, predicated upon open GWAS Catalog and FinnGen datasets, 486 serological metabolites as exposure variables and PH as the outcome variable were subjected to MR analyses, which elucidated the causal interplay between human blood metabolites and PH. The MR investigation was contingent upon the fulfillment of the following three cardinal assumptions to yield valid inferential outcomes: (1) The IV must manifest a strong correlation with blood metabolites; (2) IVs are uncorrelated with confounding variables that influence PH, such as connective tissue disease, HIV infection, portal hypertension, and congenital heart disease; (3) IVs are independently unassociated with PH. Furthermore, given the inherent low reproducibility of the MR-dependent GWAS methodology (14–16), two distinct types of PH genetic variation data were acquired, and iterative analyses were conducted to synthesize the outcomes of the dual MR studies, thereby enhancing the confidence interval of the MR estimates. The comprehensive experimental design schematic is depicted in Figure 1. STROBE-MR checklist added to Supplementary Figure S1.

### 2.2 Sources of GWAS data acquisition for pulmonary hypertension

The PH data utilized for preliminary MR analyses emanated from the FinnGen consortium R9 release dataset comprising 265,860 participants of European descent, including 234 cases and 265,626 control participants (17). The phenotype “Hypertension, Pulmonary Arterial” was incorporated in the current inquiry and has been duly corrected for 10 principal components such as age and genotyping batches. Data pertaining to PH genetic variants for replication analyses were procured from the GWAS Catalog dataset with an aggregate of 456,348 participants of European ancestry, including 99 cases and 456,249 control participants, bearing study accession “GCST90043961” in the GWAS Catalog dataset, this dataset was obtained by further analysing the UKB data through the fastGWA-GLMM (genome-wide association-generalised linear mixed model) approach (18).

The diagnostic criteria for PH, employed in both initial and replication analyses, are delineated in the International Classification of Diseases, 10th edition (ICD-10) (https://icd.who.int/browse10/2016/en#). This MR analysis was predicated on previously published articles and open-source databases and, thus, necessitated no additional ethical approval or consent.

### 2.3 Serum metabolites GWAS data source

Predicated upon the publication of So-Youn Shin, et al., we utilized genome-wide association summary datasets correlating to 486 human serum metabolites (19). This corpus of 486 metabolites comprised 177 unknown metabolites and 309 known metabolites (8 biochemical classifications, including amino acids, peptides, energy, cofactors and vitamins, lipids, xenobiotics, carbohydrates, and nucleotides). The culminating GWAS dataset summarizing approximately 2.1 million SNPs was extracted from the KORA dataset and TwinsUK dataset (7,824 adult individuals).

### 2.4 Screening for suitable instrumental variables

To amplify the reliability of the current MR findings, several iterative procedures were executed to screen qualified IVs. Initially, SNP loci exhibiting a high correlation with metabolites (*P* < 1 × 10^−5^) were isolated from the metabolite GWAS dataset; subsequently, parameters *R*^2^ = 0.01 and a genetic distance of 100 kb were implemented to ensure IV independence by eliminating linkage disequilibrium (LD) between selected IVs; ultimately, the study utilized the Fixed-effects Direction (FD) method to eradicate LD between the selected IVs. Given that the presence of weak IVs could compromise the efficacy of statistical tests and might jeopardize the foundational hypotheses, the strength of IVs was quantified via the F-statistic, and those with F-statistics less than 10 were deemed weak IVs (20, 21). The F-statistic is calculated as follows:

$$R^{2}=\frac{2\times (1-\mathrm{MAF})\times \mathrm{MAF}\times \beta }{\mathrm{SD}}$$

MAF: minor allele frequency, *β*: effect value of the allele, SD: standard error of *β*.

$$F=\frac{R^{2}\times (N-1-K)}{(1-R^{2})\times K}$$

*N*: number of samples exposed to the GWAS study, *K*: number of IVs, *R*^2^: degree to which IVs explain exposure (coefficient of determination of the regression equation).

### 2.5 Preliminary MR analysis

The PH genetic variance data utilized for the preliminary MR analysis were sourced from the FinnGen dataset. IVW functioned as the primary method of causal inference for this MR study due to its robust testing efficacy and statistical potency (22), followed by Simple mode, MR-Egger, Weighted Median, and Weighted Model as the ancillary mechanisms for causal inference. Among these, the IVW method employs a meta-analytical framework that amalgamates Wald ratio estimates of causal effects elicited from distinct SNPs and proffers a uniform evaluation of the causal effect of exposure variables on outcomes, contingent upon each genetic variant conforming to the axioms of the IVs. However, the application of IVW can engender skewed approximations of causal effects when there exists a multivariate complication with the IV (22). The MR-Egger method utilizes the regression coefficient as the causal effect approximations, but incorporates an intercept term to adjust for the potential influence of pleiotropy among IVs (23). The Weighted Model allocates proportional significance to the causal effect value of each single-nucleotide polymorphism pair with the aggregate number of single-nucleotide polymorphisms in each cluster, yielding the outcome as a provisional estimation endowed with the maximal single-nucleotide polymorphism weighting (24). The MR-Egger method utilizes the regression coefficient as the causal effect approximations, but the intercept can serve to evaluate the extent of pleiotropy among IVs.The False discovery rate (FDR) multiple hypothesis testing correction method was implemented in the preliminary and replication analyses, and we considered a significant association between metabolites and pulmonary arterial hypertension when the FDR was <0.05; however, considering that the FDR correction method is more stringent, which may lead to the occurrence of a false-negative situation, when the FDR > 0.05 and *P* < 0.05, we considered that the metabolites had a suggestive Association.

### 2.6 Sensitivity analysis

In the progression of this study, we employed the MR-Egger intercept test and the MR-PRESSO methodology to scrutinize the plethora of genetic variants. The MR-Egger intercept test calculates directional *P*-values by ascertaining the intercept to estimate directional bias (25), and the MR-PRESSO method discerns the existence of multiplicity by quantifying the heterogeneity of causal effects across diverse subgroups, which is acutely sensitive to the directional bias induced by the multiplicity of genetic variants (26). Additionally, to gauge the heterogeneity of SNPs acquired from diverse cohort studies, we assessed the potential heterogeneity between IVs in each analysis employing Cochran's Q test (27). Subsequently, the leave-one-out sensitivity test was utilized to assess whether the amalgamated IVW estimates were influenced by any individual Single Nucleotide Polymorphism (SNP) (28).

### 2.7 Metabolic pathway analysis

To investigate the potential metabolic pathways implicated in PH occurrence, the current study utilized the KEGG (Kyoto Encyclopedia of Genes and Genomes) database (https://new.metaboanalyst.ca/MetaboAnalyst/) employing a compilation of identified metabolites (*P* < 0.05). Metabolic pathway analysis was conducted using MetaboAnalyst 5.0, and subsequently, we deemed the metabolic pathways with statistically significant levels (*P* < 0.05) to be salient.

### 2.8 Exclusion of confounding factors

While the credibility of MR results was evaluated by excluding weak IVs (F < 10) and subsequent to a series of sensitivity analyses, there may exist IVs that transgress the foundational assumptions of MR (2) and (3). Consequently, we extracted the remaining IVs using PhenoScanner V2 (http://www.phenoscanner.medschl.cam.ac.uk/). We then excluded IVs correlated with confounding factors such as BMI, body fat percentage, interstitial lung disease, or factors directly pertinent to PH.

### 2.9 Mendelian randomized replication analysis and meta-analysis

To bolster the confidence level of the MR estimates, we conducted replication analyses employing an alternative independent PH genetic variation data set from the GWAS Catalog dataset (https://www.ebi.ac.uk/gwas/downloads/summary-statistics). We then amalgamated the results of the two MR analyses to identify the culminating metabolites causally linked to PH.

### 2.10 Linkage disequilibrium score regression and reverse causation analysis

Even though we excluded IVs intrinsically linked to PH, extant literature has demonstrated that combinations of IVs not overtly associated with PH may still be genetically predisposing factors for PH (28). To mitigate confounding by metabolite and PH co-inheritance, the present study employed linkage disequilibrium score regression (LDSC) to assess the genetic correlations between the candidate metabolites and PH.

In adjunction, the current study executed reverse causality MR analysis on the ascertained metabolite set to circumvent the endogeneity problem emanating from reverse causality, thereby enhancing the credibility of the extant MR results.

## 3 Results

After multiple screening steps to exclude a metabolite with fewer than 3 SNPs, 8842 SNPs associated with 485 metabolites were ultimately identified (with a minimum of 3 SNPs and a maximum of 414 SNPs). Additionally, the F-statistic of all SNPs was greater than 10, indicating all of these SNPs can serve as robust tools for MR studies. Comprehensive information is displayed in Supplementary Tables S1,S2.

### 3.1 Results of preliminary analysis of MR

**Figure 2:**
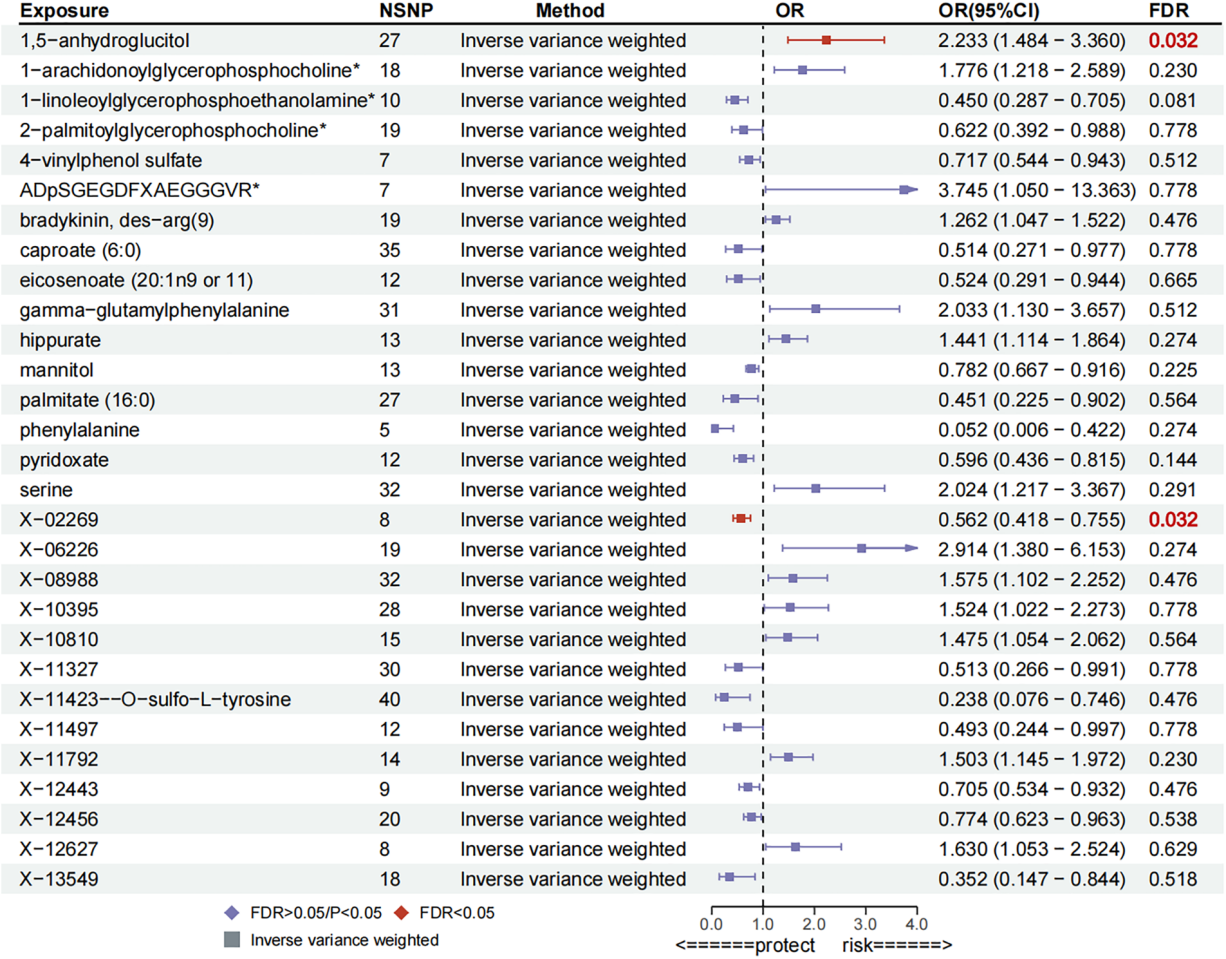
Forest plot for preliminary MR analysis.

Preliminary MR results are displayed in Supplementary Table S3. Preliminary IVW analyses indicated 29 metabolites causally associated with PH, comprising 13 serum metabolites with uncharacterized chemical properties and 16 serum metabolites with characterized biochemical properties. These metabolites were classified according to chemical properties as Carbohydrates, Lipids, Cofactors and Vitamins, Xenobiotics, Amino Acids, Peptides, and Xenobiotics. After FDR correction, a significant causal association remained between 1,5-anhydroglucitol [OR = 2.23, 95% confidence interval (CI): 1.48–3.36, *P* = 0.0001,FDR = 0.032] remained significantly and causally associated with PH, While, 1-linoleoylglycerophosphoethanolamine (OR = 0.45, 95% CI: 0.29–0.71, *P* = 0.0005, FDR = 0.081), pyridoxate (OR = 0.60, 95% CI: 0.44–0.82, *P* = 0.0012, FDR = 0.144), mannitol (OR =  0.78, 95% CI: 0.67–0.92, *P* = 0.0023, FDR = 0.225), 1-arachidonoylglycerophosphocholine (OR =  1.78, 95% CI: 1.22–2.59, *P* = 0.0029, FDR = 0.230), hippurate (OR = 1.44, 95% CI: 1.11–1.86, *P* = 0.0054, FDR = 0.274) amongst 16 other characterised metabolites with suggested causal associations with PH, details of which are illustrated in Figure 2. Moreover, certain compounds, namely X-11792, X-11423–O-sulfo-L-tyrosine, eicosenoate, ADpSGEGDFXAEGGGVR, bradykinin, and des-arg(9), exhibited evidence of heterogeneity. Additionally, X-10810-associated IVs were observed to be horizontally pleiotropic, whereas other metabolite-related IVs exhibited neither heterogeneity nor horizontal pleiotropy (Supplementary Table S4; Supplementary Figure S1).

### 3.2 Confounding analysis, replication analysis and meta-analysis

Although these 13 characterized metabolites surpassed the heterogeneity and horizontal pleiotropy tests, further scrutiny was employed to examine associations with other phenotypes of IVs correlated with these 13 metabolites. Utilizing the Phenoscanner website, associations with pyridoxate, 1-arachidonoylglycerophosphocholine, gamma-glutamylphenylalanine, 2-palmitoylglycerophosphocholine-related SNPs were found to be devoid of confounding factors affecting PH. However, we ascertained SNPs correlated with phenylalanine, palmitate, mannitol, hippurate, 1,5-anhydroglucitol, serine, caproate, 1-linoleoylglycerophosphoethanolamine, 4-vinylphenol sulfate among the SNPs associated with confounding factors such as Body Mass Index (BMI), body weight, hypertension, diastolic blood pressure, and interstitial lung disease (Supplementary Figure S1). Subsequently, these SNPs correlated with confounding variables were expunged, and post-elimination, it was determined that 1,5-anhydroglucitol, pyridoxate, mannitol, phenylalanine, serine, 2-palmitoylglycerophosphocholine, 1-arachidonoylglycerophosphocholine, gamma-glutamylphenylalanine remained causally associated with PH. Conversely, 1-linoleoylglycerophosphoethanolamine, hippurate, caproate, palmitate, 4-vinylphenol, sulfate were no longer significantly causally associated with PH.

**Figure 3:**
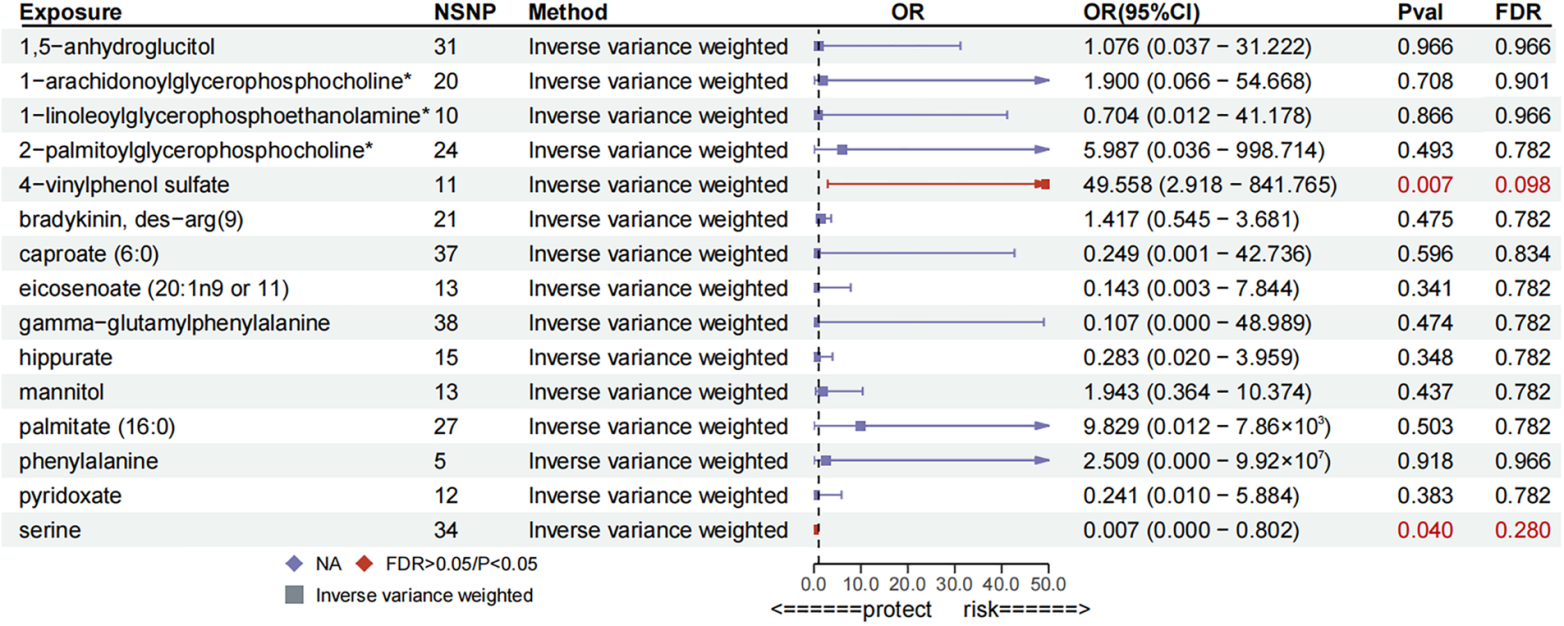
Forest plot for replication MR analysis.

**Figure 4:**
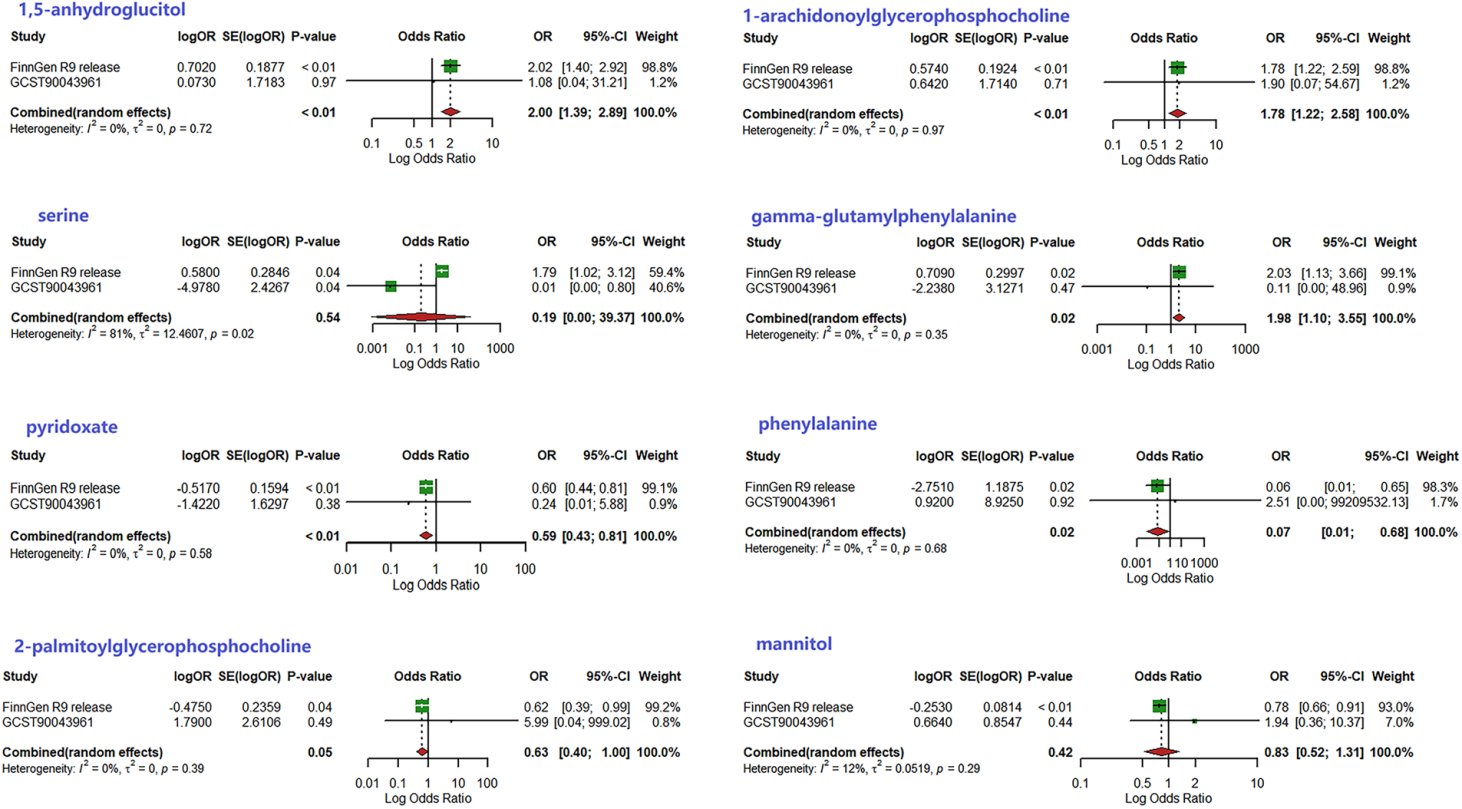
Results of meta-analysis of two MR studies.

**Figure 5:**
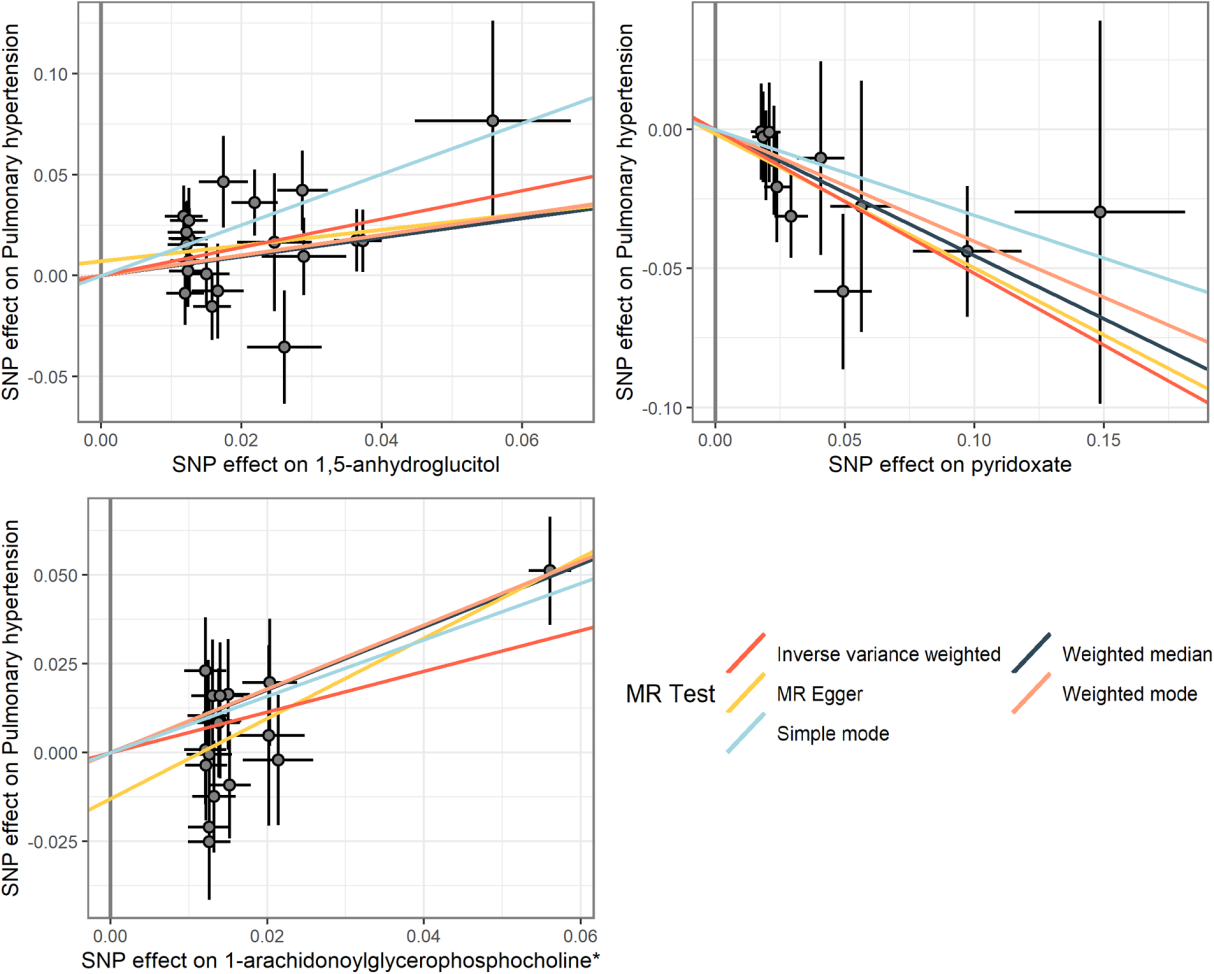
Scatterplot of MR analysis of three metabolites.

To corroborate the results of the preliminary MR analysis, independent PH genetic data were once again procured from the GWAS Catalog dataset for replication analysis. The results of the analyses replicating MR (Figure 3), the IVs used and the results of the sensitivity analyses are recorded in Supplementary Tables S5–S7, respectively.This demonstrated that three metabolites mirrored the trend observed in the preliminary analysis. The current study subsequently integrated the results of the two MR analyses into a Meta-analysis (Figure 4), which ultimately corroborated that 1,5-anhydroglucitol (OR = 2.00, 95% CI: 1.39–2.89, *P* = 0.0002), 1-arachidonoylglycerophosphocholine (OR = 1.78, 95% CI: 1.22–2.58, *P* = 0.0026), and pyridoxate (OR = 0.59, 95% CI: 0.43–0.81, *P* = 0.0009) were indeed causally associated with PH (Figure 5). Although the Meta-analysis indicated that phenylalanine (OR =  0.07, 95% CI: 0.01–0.68, *P* = 0.0224), gamma-glutamylphenylalanine (OR =  0.07, 95% CI: 0.01–0.68, *P* = 0.0224) were causally associated with PH, the two analyses did not concur in directional estimates, thereby leading to their exclusion.

### 3.3 Metabolic pathway

In the extant study, we ascertained one meaningful metabolic pathways, the Vitamin B6 metabolism pathway (*P* = 0.017, KEGG SMP), in which pyridoxate was engaged in Vitamin B6 metabolism pathway.

### 3.4 Results of LDSC and reverse causality analysis

According to the LDSC analysis, PH was not markedly genetically correlated with 1,5-anhydroglucitol (Rg = 0.054, Se = 0.101, *p* = 0.592), pyridoxate(Rg = 0.679, Se = 0.516, *p* = 0.188), pyridoxate (Rg = −0.130, Se = 0.116, *p* = 0.260). This signifies that the results of the current MR study are not obfuscated by the genetic structure concomitant between metabolites and PH.

Reverse causality evidentiality indicated that there was insufficient genetic substantiation for a causal relationship between PH and 1,5-anhydroglucitol, pyridoxate, and pyridoxate, circumventing the problem of endogeneity (Supplementary Tables S8,S9).

## 4 Discussion

In the current study, utilizing FinnGen dataset and GWAS Catalog dataset, two-sample MR analysis was employed to investigate the causal relationship between 486 human serological metabolites and PH. Subsequent to a series of rigorous validation, we ultimately found that elevated levels of 1,5-anhydroglucitol and 1-arachidonoylglycerophosphocholine augment the risk of PH, whereas elevated levels of pyridoxate mitigate the risk of PH. Additionally, we conducted LDSC analysis and ascertained that there is no genetic correlation between these three metabolites and PH, thus the results of this MR study shall not be obfuscated by co-inheritance. Finally, we identified one salient metabolic pathway, namely Vitamin B6 metabolism pathway which can facilitate our comprehension of the mechanism of PH and provide crucial insights for PH prevention and pharmaceutical development.

The detriment of PH has imposed a heavy burden on society; thus, the prevention and treatment of PH constitute a clinical imperative. Extant data imply that PH may be correlated with certain genetic mutations, connective tissue diseases (e.g., systemic lupus erythematosus, scleroderma), HIV infection, and congenital heart disease (29); however, the etiology of pulmonary arterial hypertension remains incompletely elucidated. Serological markers that have been correlated with pulmonary arterial hypertension comprise Cystatin C, endothelin, C-reaction protein, and inflammatory mediators (30–32). The current evidence has not yet definitively ascertained the causal role of serum metabolites in the development of PH. In this study, we conducted an MR analysis predicated on the GWAS dataset to comprehensively discern the causal relationship between circulating metabolites and PH.

The current MR study suggests that elevated levels of 1,5-anhydroglucitol and 1-arachidonoylglycerophosphocholine (1-AGPC) may connote an elevated risk of developing PH. Nonetheless, there exist no investigations delineating a direct causal relationship between 1,5-anhydroglucitol and PH, and research concerning the importance of 1,5-anhydroglucitol markers remains ambivalent. Some studies indicate that low levels of 1,5-anhydroglucitol serve as a biomarker for diabetes and cardiovascular disease (33, 34), whereas other studies have demonstrated that serum levels of 1,5-anhydroglucitol are markedly elevated during glucose tolerance tests (35). Additional studies contend that serum 1,5-anhydroglucitol levels are not significantly associated with blood glucose and that patients with cirrhosis exhibit a significant increment in 1,5-anhydroglucitol levels in liver tissue and a diminution in serum 1,5-anhydroglucitol levels (36). The aforementioned studies lack multicenter, large-scale cohort studies and are constrained by methodological limitations. The current study utilized MR studies to circumvent traditional methodological constraints, revealing that elevated levels of 1,5-anhydroglucitol may contribute to the pathogenesis of PH, which proffers a novel trajectory for more in-depth investigations in the future.

In addition, the current study identified elevated levels of 1-AGPC as a risk factor for PH, yet extant literature on the causal nexus between 1-AGPC and PH is sparse. 1-AGPC is a lipid molecule ubiquitous in the human physiological matrix, which, upon metabolism, generates bioactive moieties such as arachidonic acid (AA). Existing studies demonstrate that AA is transformed into a spectrum of intracorporeal metabolites, including prostaglandins and leukotrienes, that are hypothesized to exhibit a correlation with hypertension (29). These metabolites exert functions such as inducing vasoconstriction, augmenting blood coagulation, and initiating inflammation. These effects may harbor relevance to the development and progression of PH, thus bolstering the conclusions of the present MR study.

This MR study substantiates the protective efficacy of pyridoxate against PH, a claim corroborated by antecedent research. Pyridoxate constitutes the hydrochloride isomer of vitamin B6, which is biochemically transmuted into its active form *in vivo*. Evidential support exists for the notion that vitamin B6 mitigates blood homocysteine concentrations, thereby attenuating cardiovascular risk (37). In contemporary investigations, an inverse prevalence of hypertension has been associated with elevated consumption of vitamin B6 (38, 39), and it has been elucidated that pyridoxate modulates cellular calcium transport via voltage- and ATP-mediated purinergic endocytic mechanisms, thereby serving a regulatory function in hypertension management (40). Another cross-sectional inquiry involving 2,241 adults in rural China indicated that elevated vitamin B-6 intake correlates independently with a diminished risk of hypertension (41).

The present MR study boasts several merits: (1) IVs we scrutinized and the exposures manifested robust correlation (F-statistic >10); this MR study utilized polymorphic data related to PH from divergent repositories, executed repetitive analyses, and amalgamated the outcomes of the two MR studies, thereby enhancing the reliability of the MR estimation; (2) the onerous task of accruing PH-related cases in clinical settings renders observational studies particularly challenging to execute, and complicates the mitigation of confounding variables; (3) MR studies outperform conventional observational methodologies in feasibility and result robustness; (4) this research undertook a reverse causality investigation to circumvent endogeneity-related pitfalls.

However, this MR study also exhibits intrinsic limitations: firstly, we sourced GWAS data exclusively from European cohorts, rendering the MR findings somewhat parochially represented; secondly, albeit the employed MR serves as a potent instrument for causal inference, its conclusions necessitate corroboration via ensuing empirical data; and lastly, due to laboratory and practical constraints, this study did not involve hematological testing of animal or human samples, but the MR study was able to give some causal analysis of associations at the GWAS level, which is a major drawback of this study, and there will be opportunities to further supplement this portion of the trial in the future.

## 5 Conclusions

In summation, we employed a multifaceted analytical approach to isolate three metabolites from a compilation of 486 serum metabolites for putative causal association with PH, including 1,5-anhydroglucitol, 1-AGPC, and Pyridoxate. This furnishes an evidential framework for the formulation of future strategies for early diagnostic assessment and prophylaxis of PH, bearing significant ramifications for clinical practice.

## Data Availability

Publicly available datasets were analyzed in this study. This data can be found here: human blood metabolites: https://pubmed.ncbi.nlm.nih.gov/24816252/; pulmonary hypertension: FinnGen consortium R9 and http://ftp.ebi.ac.uk/pub/databases/gwas/summary_statistics/GCST90043001-GCST90044000/GCST90043961/.
