# Supplementary material for: Investigating the causal relationship between human blood metabolites and pulmonary hypertension: a two-sample Mendelian randomization study
Source: Front Cardiovasc Med. 2024 Oct 15;11:1304986. doi: 10.3389/fcvm.2024.1304986 (PMC11518716; doi:10.3389/fcvm.2024.1304986)
Supplement: Supplementary file 2 [file Datasheet1.docx]

**STROBE-MR checklist of recommended items to address in reports of Mendelian randomization studies**^1^ ^2^

| **Item No.** | **Section** | **Checklist item** | **Page No.** | **Relevant text from manuscript** |
| --- | --- | --- | --- | --- |
| 1 | **TITLE and ABSTRACT** | Indicate Mendelian randomization (MR) as the study’s design in the title and/or the abstract if that is a main purpose of the study | 1 | A two-sample Mendelian randomization study was conducted to investigate the causal effects of serum metabolites on pulmonary hypertension. |
|  | **INTRODUCTION** |  |  |  |
| 2 | **Background** | Explain the scientific background and rationale for the reported study. What is the exposure? Is a potential causal relationship between exposure and outcome plausible? Justify why MR is a helpful method to address the study question | 1-2 | Several recent investigations have posited that distinct metabolites in the bloodstream may be correlated with the pathogenesis of Pulmonary Hypertension (PH). Nonetheless, the interrelationship between the pathogenesis of PH and metabolite fluctuations remains incompletely  elucidated, and findings may differ across studies. |
| 3 | **Objectives** | State specific objectives clearly, including pre-specified causal hypotheses (if any). State that MR is a method that, under specific assumptions, intends to estimate causal effects | 1 | We conducted a Mendelian randomization analysis to assess the causal relationship between human blood metabolites and susceptibility to pulmonary hypertension. |
|  | **METHODS** |  |  |  |
| 4 | **Study design and data sources** | Present key elements of the study design early in the article. Consider including a table listing sources of data for all phases of the study. For each data source contributing to the analysis, describe the following: |  |  |
|  | a) | Setting: Describe the study design and the underlying population, if possible. Describe the setting, locations, and relevant dates, including periods of recruitment, exposure, follow-up, and data collection, when available. | 2-3 | Figure 1 provides a schematic summary of the study design.  The sources of data on exposure and outcomes are described in the original "2.2 Sources of GWAS data acquisition for pulmonary hypertension"and "2.3 Serum metabolites GWAS data source"section.  Cases and controls were defined based on ICD10-codes |
|  | b) | Participants: Give the eligibility criteria, and the sources and methods of selection of participants. Report the sample size, and whether any power or sample size calculations were carried out prior to the main analysis | 3 | Exposure: The comprehensive summary statistics of genetic influence on human serum metabolites in the TwinsUK and KORA studies provide extensive data for GWAS on the human metabolome. The dataset includes genome-wide genotyping data from 7,824 European participants, and a total of 486 metabolite concentrations were tested in the GWAS  Outcome:In the section "2.2 Sources of GWAS data acquisition for pulmonary hypertension" in the original article. |
|  | c) | Describe measurement, quality control and selection of genetic variants | 3 | The lowest F-statistic value found in the validity test exceeded the threshold of 10,indicating a negligible probability of encountering weak instrument bias . Only independent SNPs (r^2^= 0.01; distance = 100 kb), strongly associated (P ≤ 1 × 10^-5^) with the blood level of each metabolite, were used in the primary analyses |
|  | d) | For each exposure, outcome, and other relevant variables, describe methods of assessment and diagnostic criteria for diseases |  | Exposure: A total of 486 metabolites.  Outcome: Cases and controls were defined based on ICD10-codes. |
|  | e) | Provide details of ethics committee approval and participant informed consent, if relevant |  | The studies in these consortia obtained approval from local research ethics committees and institutional review boards, and all participants provided written informed consent. |
| 5 | **Assumptions** | Explicitly state the three core IV assumptions for the main analysis (relevance, independence and exclusion restriction) as well assumptions for any additional or sensitivity analysis | 4 | Three necessary assumptions were explained.We employed MR-Egger method, weighted median analysis, and MR-PRESSO test as sensitivity analysis methods |
| 6 | **Statistical methods: main analysis** | Describe statistical methods and statistics used |  |  |
|  | a) | Describe how quantitative variables were handled in the analyses (i.e., scale, units, model) | NA |  |
|  | b) | Describe how genetic variants were handled in the analyses and, if applicable, how their weights were selected | NA |  |
|  | c) | Describe the MR estimator (e.g. two-stage least squares, Wald ratio) and related statistics. Detail the included covariates and, in case of two-sample MR, whether the same covariate set was used for adjustment in the two samples | 4 | IVW functioned as the primary method of causal inference for this MR study due to its robust testing efficacy and statistical potency |
|  | d) | Explain how missing data were addressed |  | After multiple screening steps to exclude a metabolite with fewer than 3 SNPs, 8,842 SNPs associated with 485 metabolites were ultimately identified (with a minimum of 3 SNPs and a maximum of 414 SNPs). |
|  | e) | If applicable, indicate how multiple testing was addressed | NA |  |
| 7 | **Assessment of assumptions** | Describe any methods or prior knowledge used to assess the assumptions or justify their validity | 3 | Only independent SNPs (r^2^= 0.01; distance = 100 kb), strongly associated (P ≤ 1 × 10^-5^) with the blood level of each serum metabolites. |
| 8 | **Sensitivity analyses and additional analyses** | Describe any sensitivity analyses or additional analyses performed (e.g. comparison of effect estimates from different approaches, independent replication, bias analytic techniques, validation of instruments, simulations) | 4 | We conducted sensitivity analyses to account for pleiotropy for genetic instruments with ≥ 3 variants: MR Egger, Cochran's Q test and MR-PRESSO. |
| 9 | **Software and pre-registration** |  |  |  |
|  | a) | Name statistical software and package(s), including version and settings used | 4 | The statistical analyses were performed using R software version 4.2.3. The MR analyses were performed using the TwoSampleMR package and the MRPRESSO package.  Phenoscanner, available at http://www.phenoscanner.medschl.cam.ac.uk/, was used to evaluate whether the association could be due to pleiotropy. |
|  | b) | State whether the study protocol and details were pre-registered (as well as when and where) |  | NO |
|  | **RESULTS** |  |  |  |
| 10 | **Descriptive data** |  |  |  |
|  | a) | Report the numbers of individuals at each stage of included studies and reasons for exclusion. Consider use of a flow diagram |  | Figure 1 provides a schematic summary of the study design. |
|  | b) | Report summary statistics for phenotypic exposure(s), outcome(s), and other relevant variables (e.g. means, SDs, proportions) | 2-3 | The sources of data on exposure and outcomes are described in the original "2.2 Sources of GWAS data acquisition for pulmonary hypertension"and "2.3 Serum metabolites GWAS data source"section. |
|  | c) | If the data sources include meta-analyses of previous studies, provide the assessments of heterogeneity across these studies |  |  |
|  | d) | For two-sample MR:  i.  Provide justification of the similarity of the genetic variant-exposure associations between the exposure and outcome samples  ii.  Provide information on the number of individuals who overlap between the exposure and outcome studies |  | i: We used different data sources for exposures and outcomes. We calculated the Cochran's Q-test for heterogeneity to assess heterogeneity across the cohorts and found minimal heterogeneity for the included variants for the outcomes  ii: Because we used summary-level statistics, we couldn't identify individuals who were common to both the exposure and outcome datasets. |
| 11 | **Main results** |  |  |  |
|  | a) | Report the associations between genetic variant and exposure, and between genetic variant and outcome, preferably on an interpretable scale | 3 | Using r2 < 0.01 within 100 kb windows and P ≤ 1E-05. |
|  | b) | Report MR estimates of the relationship between exposure and outcome, and the measures of uncertainty from the MR analysis, on an interpretable scale, such as odds ratio or relative risk per SD difference |  | OR |
|  | c) | If relevant, consider translating estimates of relative risk into absolute risk for a meaningful time period | NA |  |
|  | d) | Consider plots to visualize results (e.g. forest plot, scatterplot of associations between genetic variants and outcome versus between genetic variants and exposure) |  | Figure 2 and 4. |
| 12 | **Assessment of assumptions** |  |  |  |
|  | a) | Report the assessment of the validity of the assumptions | 4-5 | Methods to assess the robustness of MR findings: MR Egger, MRPRESSO, Cochran's Q statistical test, PhenoScanner, leave-one-out analyses and reverse MR analysis. |
|  | b) | Report any additional statistics (e.g., assessments of heterogeneity across genetic variants, such as *I^2^*, Q statistic or E-value) | 5 | Q-test were used to assess potential heterogeneity and identify outliers in the IVW and MR-Egger analyses. |
| 13 | **Sensitivity analyses and additional analyses** |  |  |  |
|  | a) | Report any sensitivity analyses to assess the robustness of the main results to violations of the assumptions | 5 | Table S4 for the MR analyses. |
|  | b) | Report results from other sensitivity analyses or additional analyses | 5 | Table S4 |
|  | c) | Report any assessment of direction of causal relationship (e.g., bidirectional MR) | 6 | We conducted a bidirectional two-sample MR analysis using blood levels of metabolites as the exposure and pulmonary hypertension as the outcome(Additional Table S5,S6). |
|  | d) | When relevant, report and compare with estimates from non-MR analyses |  |  |
|  | e) | Consider additional plots to visualize results (e.g., leave-one-out analyses) |  | Leave-one-out results are presented in Additional Document 2. |
|  | **DISCUSSION** |  |  |  |
| 14 | **Key results** | Summarize key results with reference to study objectives | 6 | In the current study, utilizing GWAS dataset and FinnGen dataset, two-sample MR analysis was employed to investigate the causal relationship between 486 human serological metabolites and PH. Subsequent to a series of rigorous validation, we ultimately found that elevated levels of 1,5-anhydroglucitol and 1-arachidonoylglycerophosphocholine augment the risk of PH, whereas elevated levels of pyridoxate mitigate the risk of PH |
| 15 | **Limitations** | Discuss limitations of the study, taking into account the validity of the IV assumptions, other sources of potential bias, and imprecision. Discuss both direction and magnitude of any potential bias and any efforts to address them | 8 |  |
| 16 | **Interpretation** |  |  |  |
|  | a) | Meaning: Give a cautious overall interpretation of results in the context of their limitations and in comparison with other studies | 7-8 | Given the significant role of metabolites in the development and maintenance of the hypertension, it is biologically plausible that certain metabolites may influence the risk of pulmonary hypertension. Based on our findings, this provides early predictive factors for future research on the utility of these biomarkers in blood tests for pulmonary hypertension prevention. |
|  | b) | Mechanism: Discuss underlying biological mechanisms that could drive a potential causal relationship between the investigated exposure and the outcome, and whether the gene-environment equivalence assumption is reasonable. Use causal language carefully, clarifying that IV estimates may provide causal effects only under certain assumptions | 7 |  |
|  | c) | Clinical relevance: Discuss whether the results have clinical or public policy relevance, and to what extent they inform effect sizes of possible interventions | 7-8 |  |
| 17 | **Generalizability** | Discuss the generalizability of the study results (a) to other populations, (b) across other exposure periods/timings, and (c) across other levels of exposure |  |  |
|  | **OTHER INFORMATION** |  |  |  |
| 18 | **Funding** | Describe sources of funding and the role of funders in the present study and, if applicable, sources of funding for the databases and original study or studies on which the present study is based |  | The present research utilized publicly available summary data, for which no additional ethical approval was required. This study did not receive any funding. |
| 19 | **Data and data sharing** | Provide the data used to perform all analyses or report where and how the data can be accessed, and reference these sources in the article. Provide the statistical code needed to reproduce the results in the article, or report whether the code is publicly accessible and if so, where | 3 | In the section "2.2 Sources of GWAS data acquisition for pulmonary hypertension" in the original article. |
| 20 | **Conflicts of Interest** | All authors should declare all potential conflicts of interest | 8 | All authors declare that the research was conducted in the absence of any commercial or financial relationships that could be construed as a potential conflict of interest. |

This checklist is copyrighted by the Equator Network under the Creative Commons Attribution 3.0 Unported (CC BY 3.0) license.

1. Skrivankova VW, Richmond RC, Woolf BAR, Yarmolinsky J, Davies NM, Swanson SA, et al. Strengthening the Reporting of Observational Studies in Epidemiology using Mendelian Randomization (STROBE-MR) Statement. JAMA. 2021;under review.

2. Skrivankova VW, Richmond RC, Woolf BAR, Davies NM, Swanson SA, VanderWeele TJ, et al. Strengthening the Reporting of Observational Studies in Epidemiology using Mendelian Randomisation (STROBE-MR): Explanation and Elaboration. BMJ. 2021;375:n2233.
