# Supplementary material for: Investigating the causal relationship between human blood metabolites and pulmonary hypertension: a two-sample Mendelian randomization study
Source: Front Cardiovasc Med. 2024 Oct 15;11:1304986. doi: 10.3389/fcvm.2024.1304986 (PMC11518716; doi:10.3389/fcvm.2024.1304986)
Supplement: Supplementary file 3 [file Datasheet2.docx]

**Elimination of confounding factors**

Among the SNPs associated with **phenylalanine**, among which 1 SNP**(rs2905873)** was also associated with BMI;

Among the SNPs associated with **palmitate**, among which 2 SNPs **(rs13355979,rs6540294)** were also associated with hypertension;

Among the SNPs associated with **mannitol**, among which 1 SNP**(rs11487092)** was also associated with BMI;

Among the SNPs associated with **hippurate**,among which 2 SNPs **(rs1878105,rs475322)** were also associated with body weight and BMI;

Among the related SNPs associated with 1**,5-anhydroglucitol,** among which 3 SNPs**(rs12603532, rs214247,rs4556997)** were also associated with body weight;

Among the SNPs associated with **serine**, among which 3 SNPs **(rs1042725,rs2762343,rs715)** were also associated with body weight and BMI;

Among the SNPs associated with **caproate**, among which 2 SNPs **(rs6498908, rs6711493)** were also associated with diastolic blood pressure;

Among the SNPs associated with **1-linoleoylglycerophosphoethanolamine,** among which 2 SNPs**(rs174535,rs551488)** were also associated with BMI and interstitial lung disease;

Among the SNPs associated with **4-vinylphenol sulfate** SNPs, among which 1 SNP**(rs1165165)** was also associated with BMI.
