# Supplementary material for: Investigating the causal relationship between human blood metabolites and pulmonary hypertension: a two-sample Mendelian randomization study
Source: Front Cardiovasc Med. 2024 Oct 15;11:1304986. doi: 10.3389/fcvm.2024.1304986 (PMC11518716; doi:10.3389/fcvm.2024.1304986)
Supplement: Supplementary file 4 [file Image1.pdf]

Supplementary Figure 1. Leave-one-out plots for the causal association between human blood metabolites and pulmonary hypertension

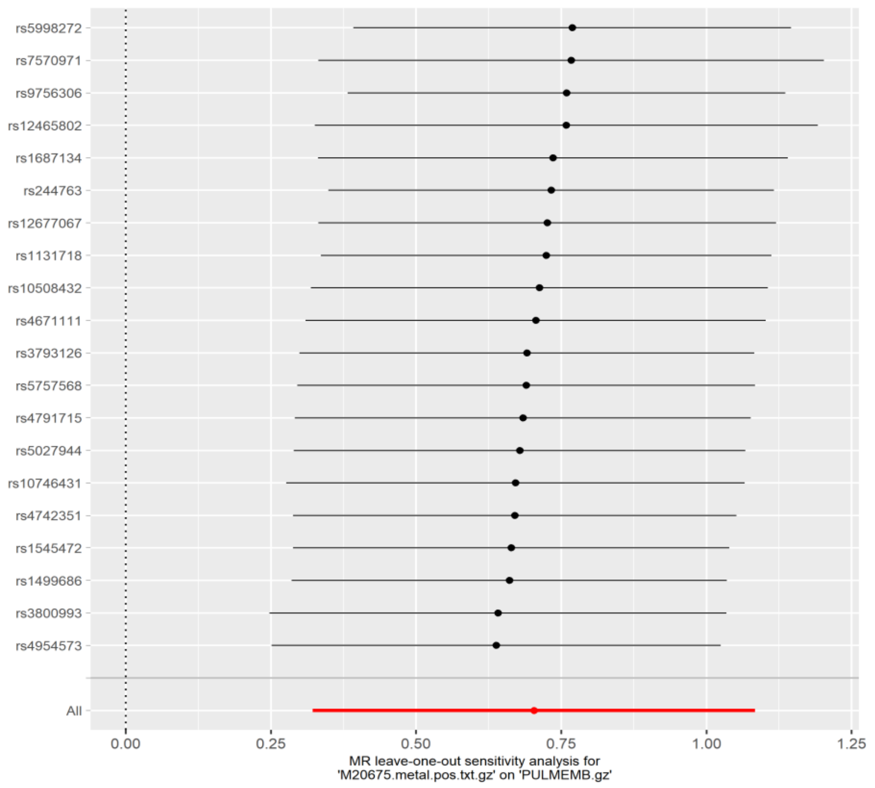

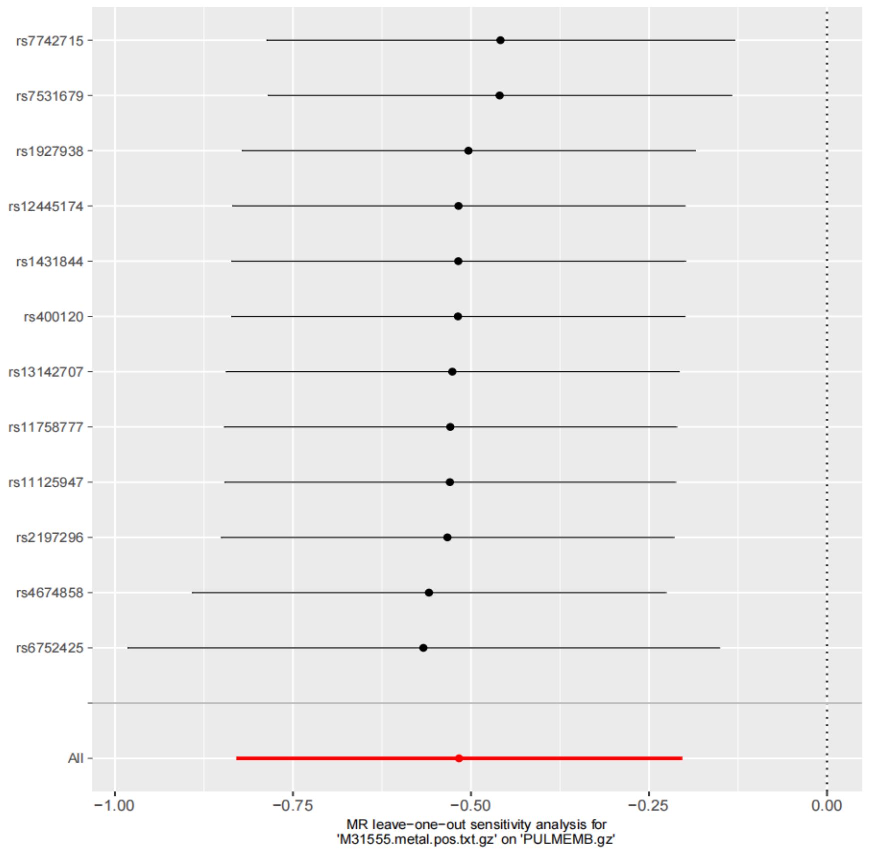

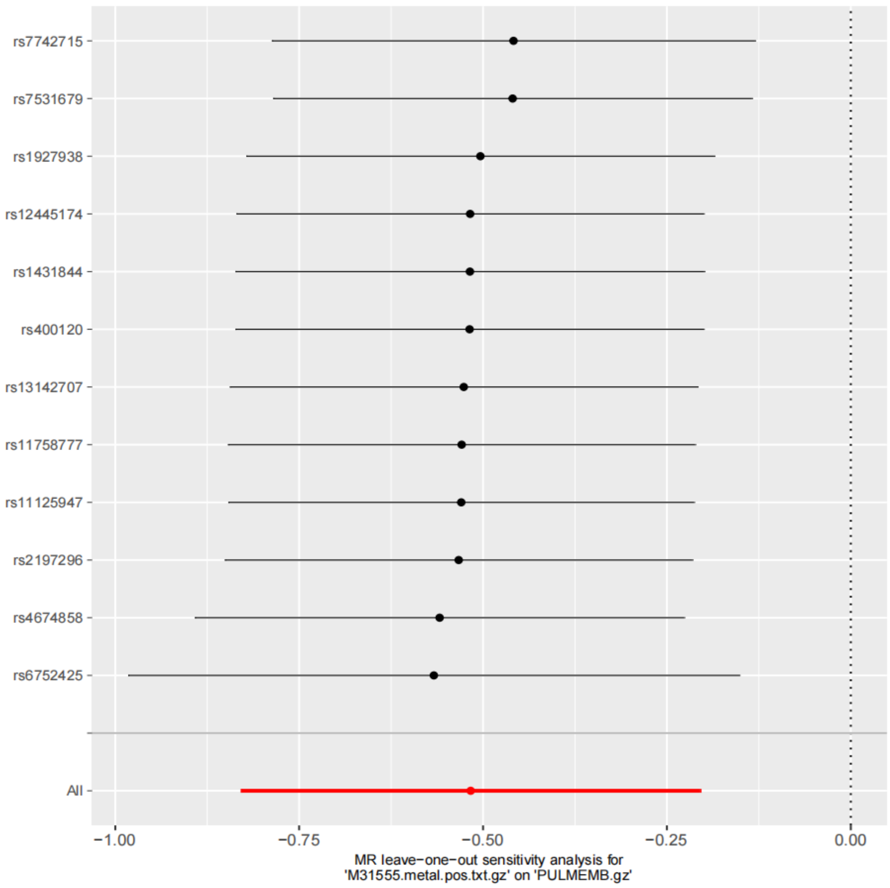
rs7742715
rs7531679
rs1927938
rs12445174
rs1431844
rs400120
rs13142707
rs11758777
rs11125947
rs2197296
rs4674858
rs6752425
All
−1.00
−0.75
−0.50
−0.25
0.00
MR leave−one−out sensitivity analysis for
'M31555.metal.pos.txt.gz' on 'PULMEMB.gz'
